# Creation of a Proof-of-Concept 3D-Printed Spinal Lateral Access Simulator

**DOI:** 10.7759/cureus.25448

**Published:** 2022-05-29

**Authors:** Michael W Pullen, Fidel Valero-Moreno, Suraj Rajendran, Vishal U Shah, Beau R Bruneau, Jaime L Martinez, Andres Ramos-Fresnedo, Alfredo Quinones-Hinojosa, W. Christopher Fox

**Affiliations:** 1 Neurological Surgery, Mayo Clinic, Jacksonville, USA; 2 Biomedical Engineering, Georgia Institute of Technology, Atlanta, USA; 3 Neurosurgery, Medical University of South Carolina, Charleston, USA

**Keywords:** ‎3d printing, lumbar interbody fusion, lumbar spine surgery, medical education and simulation, mis llif, three-dimensional (3d) printing

## Abstract

Background

Minimally invasive lateral lumbar interbody fusion (LLIF) offers advantages over traditional approaches, providing indirect decompression of neural elements and deformity correction while avoiding many challenges and risks of anterior and posterior approaches. Mastering this technique requires a specialized team, advanced equipment, and sufficient case exposure. Current training is limited to the classic educational model, and alternative training methods such as cadaver labs can be inconvenient, inaccessible, expensive, and incompatible with intraoperative neuromonitoring (IONM) systems.

Objective

The aim of this study was to create a proof-of-concept, low-cost, fully synthetic lateral lumbar surgical simulator and to increase awareness of the lack of current training alternatives.

Methods

Standard engineering design and expert interviews of attending neurosurgeons, nurses, engineers, and medical device representatives (n=20) were utilized to determine key elements for the simulator, physical characteristics of the components, and translational strategy. Physical and radiographic testing was performed on multiple thermoplastics to determine appropriateness for inclusion in the simulator. For evaluation of the concept, a descriptive slide deck and questionnaire were sent to 15 U.S. and 15 international surgeons who perform LLIF.

Results

The lateral access training model (LATM) features the following three components: torso casing, spine module, and IONM feature. This model utilizes operable ABS (acrylonitrile butadiene styrene) 3D-printed lumbar vertebrae, verified for anatomical accuracy and compatibility with fluoroscopy. Additionally, a novel neuromonitoring simulation algorithm was developed to train junior residents on neurological complications. To further highlight the need for lateral training models, 30/30 polled surgeons felt that this simulator has value for the field, 29/30 noted that they would have used the LATM if they had access during training, and 30/30 responded that they would encourage trainees to practice on the LATM.

Conclusion

The LATM is a first step to provide reliable and inexpensive basic lateral lumbar spine training. While this model is lacking some anatomical features, our simulator offers novel training elements for lateral lumbar transpsoas approaches, which lay the foundation for future models to be built. The need for this training exists, and current gaps in the approach to learning these complex techniques need to be filled due to the inconvenience, cost, and impracticability of standard cadaveric models.

## Introduction

One of the first descriptions of minimally invasive anterior approaches to the lumbar spine was presented by McAfee more than 20 years ago [1]. Following this, Ozgur et al. described the lateral transpsoas approach, which allowed surgeons to reduce the risk of significant complications, such as major vessel injury, and place large interbody grafts given the parallel approach to the disc space, all without the need for an approach surgeon [2]. The lateral approach to the lumbar column is intended to provide indirect decompression of the neural structures through restoration of the collapsed disk space and improve alignment while preserving the paraspinal muscles and the posterior elements. Currently, lateral lumbar interbody fusion (LLIF) represents a well-established surgical procedure and has become widely adopted by a subset of spine surgeons for the treatment of degenerative conditions [3-5].

Acquiring confidence with this technique requires special training and a steep learning curve that may be limited by inadequate case exposure, lack of highly specialized equipment (including EMG monitoring), and more recently the reduction of elective cases during the peaks of the COVID-19 pandemic. Three-dimensional printing and surgical simulators provide a means for reducing the learning curve, increasing resident and fellow autonomy, allowing presurgical practice before complex cases, and ultimately improving patient outcomes.

Recently, simulation devices have proven their potential to create a unique surgical environment that allows residents to operate in realistic conditions and, most importantly, to transfer new skills to the operating room [6-9]. However, no simulation tool is currently available (or able) to replicate the anatomical and technical nuances of the minimally invasive lateral retroperitoneal transpsoas approach to the lumbar spine; acquisition of new skills and performance improvement remain bound to the hospital setting and the classic teaching method. In this article, we introduce a proof-of-concept surgical simulator for lateral access spinal surgery and lay the framework for further innovation.

## Materials and methods

To determine training and design needs, interviews were conducted with neurosurgeons, engineers, nurses, and industry partners (n=20) at the Mayo Clinic, Emory University, and Georgia Institute of Technology: nine neurosurgeons, five engineers, two nurses, and four medical device industry partners. The individuals interviewed had a thorough understanding of the LLIF procedure and related subject matter. Through these interviews, we determined that there is an established need for alternatives to the classic teaching method of cadaver training for lateral access surgery. Interviewees agreed on critical inputs that a solution must have to provide proper visual, tactile, and auditory feedback.

The ideation process for the simulator began with sketch generation that was directed by the skills and knowledge intended to develop in the trainee. The concept creation process focused on specific learning objectives: safely approaching the lateral lumbar spine, identifying and avoiding excess lumbar plexus retraction, and performing a successful discectomy and placement of an interbody device. A total of 76 potential design sketches were created, which, upon end-user review, resulted in nine unique ideas. The nine designs were converted into three subcategories: the housing, the spine module, and the neuromonitoring training component. The top design concept from each category was determined using decision matrices. The top scoring concepts from each category were selected for the final design.

The proposed design was a synthetic torso that was operable, synthetic, inexpensive, and compatible with common surgical instruments and fluoroscopy. After careful analysis of the design inputs and critical product features, the lateral access training model (LATM) was created. The LATM features three major components: the outer casing, the spine module, and the intraoperative neuromonitoring (IONM) feature.

Validation of model translation

Due to the COVID-19 pandemic, in-person physician testing was not possible. In lieu of this, a descriptive slide deck was sent to surgeons who perform the LLIF procedure. This slide deck overviewed the casing, spine module, and IONM components of the device. Surgeons were then asked on the last slide to complete an anonymous three-question survey, as well as leave any feedback. The survey questions were as follows:

1. Do you feel this simulator can be valuable to the field?

2. Would you have used this simulator if you had access during your training?

3. Would you encourage residents or medical students to train on such a simulator?

## Results

Housing

**Figure 1:**
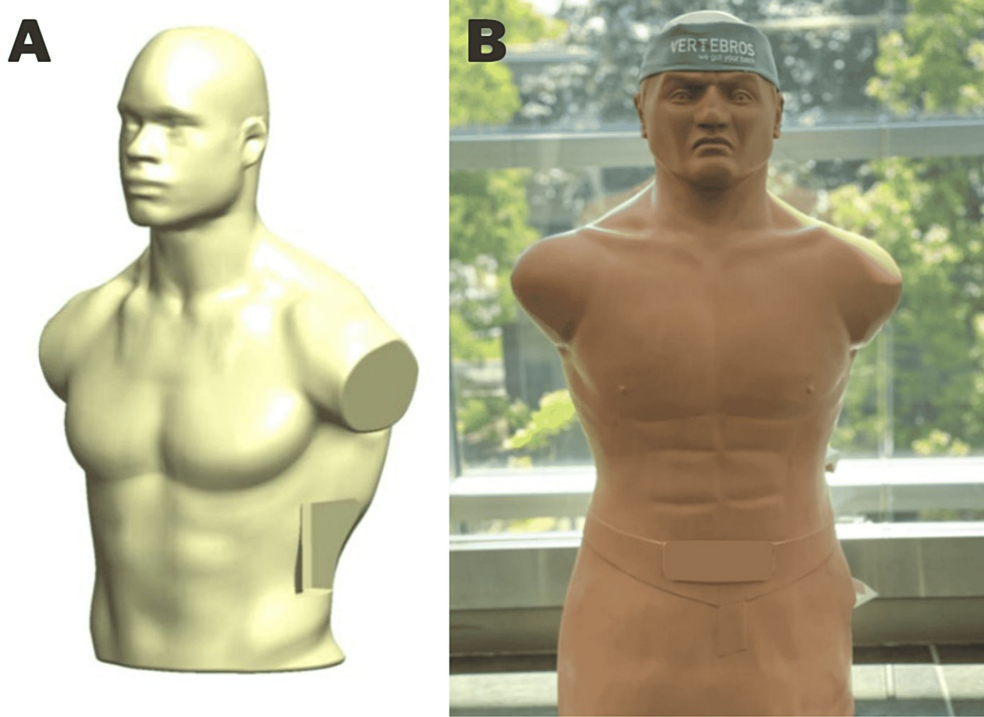
(A) Computer-aided design rendering of synthetic silicone and polymer foam torso housing. (B) Silicone and polymer foam torso shell were used in the construction of the lateral access training model.

**Figure 2:**
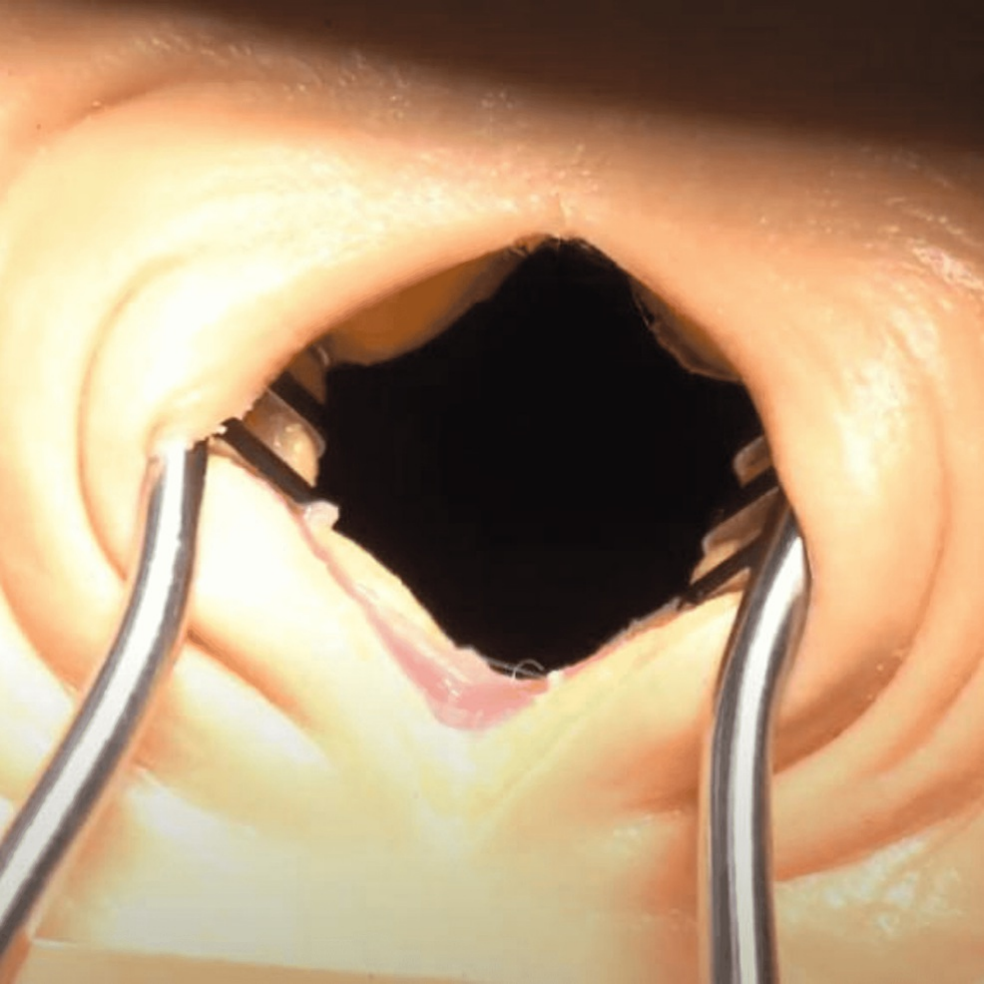
Synthetic tissue being tested (2” incision) with common surgical scalpels and retractors in a simulated operating room.

The first requirement addressed was the outer torso shell. A synthetic torso was comprised of thermoplastic foam and silicone was chosen to mimic a patient silhouette in the OR (Figures 1A, 1B). The dimensions of the torso were 30” x 20” x 10”. The attributes of the silicone housing enable realistic surgical positioning on the “broken” operating table between 190 and 225 degrees [2]. This synthetic device also provides a realistic operative field depth, which cannot be applied in box simulators. The torso allows for practice of traditional surgical site draping, positioning, and operative table setup. An interchangeable synthetic fiber-based tissue pad was included in the surgical site to mimic the abdominal wall (Figure 2). The tissue pad was held flush with the surrounding silicone torso skin with a barbed PLA (polylactic acid) 3D-printed housing creating a 7” x 3” surgical site. The housing was not visible once the surgical site was prepped.

Spine module

**Figure 3:**
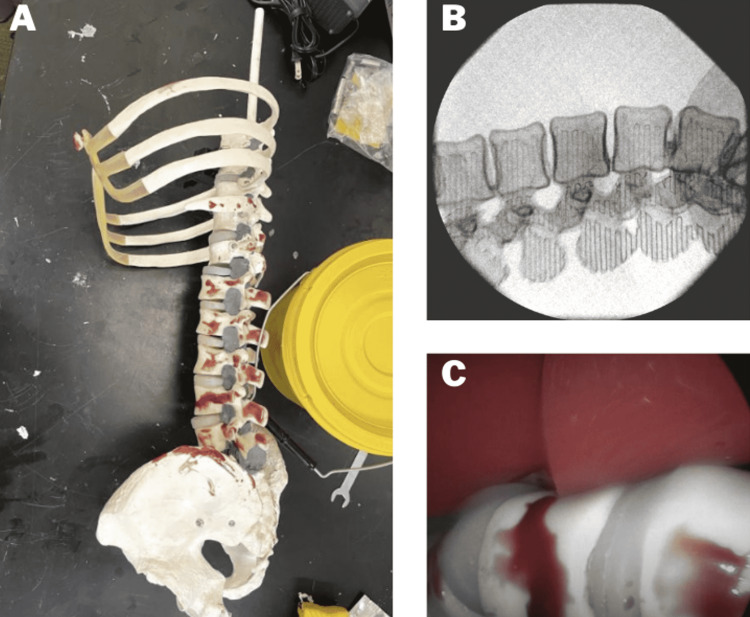
(A) Assembled spinal module consisting of PVC pelvis and sacrum, 3D-printed ABS L1-L5, silicone discs, and PVC T8-T12 vertebrae with partial rib cage. (B) Lateral fluoroscopic imaging of a 3D-printed ABS L1-L5 model including the iliac crest. (C) Laparoscopic image of synthetic psoas muscle atop of L2-L3 3D-printed vertebrae with silicone intervertebral discs. PVC, polyvinyl chloride; ABS, acrylonitrile butadiene styrene

The LLIF spinal column module consisted of a polyvinyl chloride (PVC) pelvis, operable acrylonitrile butadiene styrene (ABS) 3D-printed L1-L5 vertebrae, removable silicone discs, and PVC thoracic spine and partial rib cage, all secured via two-part epoxy to supportive ¼” polymer tube (Figure 3A). Files for creating the 3D-printed vertebrae were downloaded from Thingiverse (MakerBot Industries, New York, NY), a website providing open-source hardware designs. The vertebrae were scaled and perforated to allow for optimal integration into the overall spine module. An infill of 25% was used to eliminate unnecessary material and mimic the cortico-cancellous interface, as validated by prior work [10]. The vertebrae STL files were uploaded to Ultimaker Cura (Ultimaker B.V., Utrecht, the Netherlands), an open-source 3D printing software, and individually printed on the Stratasys F170 industrial printer (Stratasys Ltd, Rehovot, Israel). STL or stereolithography files are a common file type outputted by computer-aided design software and compatible with 3D print slicer programs [11].

The radiopacity of the spine was a critical need for the LATM. Through radiographic testing, it was determined that ABS 3D-printed vertebrae were best suited to use as a human analog (Figure 3B). The advantage of using 3D-printed ABS is that it performs well with spinal instrumentation. Common “classroom” spinal models are anchored with a metal rod inserted through the vertebral body. This type of structure compromises the operability and realism of the model and interferes with fluoroscopic imaging. The LATM secures individual vertebrae and pelvis with a flexible polymer tube housed within the spinal canal. The thecal sac and nervous tissue sit within the vertebral canal; therefore, having a support structure in this area does not interfere with the operability of the model as the spinal canal should never be violated during LLIF. The selected polymer support has an element of flexibility that allows the spine to preserve its natural lumbar lordosis [12]. A mixture of red-pigmented paint and clear two-part epoxy was added to the completed module to mimic residual anatomical tissues.

In this procedure, landmarks such as the iliac crest and 12th rib are used to identify the surgical site. The proposed model includes structures spanning T8 through the pelvis. Including anatomy only pertinent to the procedure minimizes total raw materials and associated costs [13]. An interchangeable synthetic fiber-based psoas muscle (11.2” x 2.8” x 2.9”) was anchored within the spine module to allow for dilation training (Figure 3C). The synthetic psoas was held in place by heavy-duty tarp clips that were anchored on the pelvis and thoracic spine with self-tapping eyelet screws. The housing and clip system for the abdominal tissue pad and the psoas muscle allows for reusability of the module. The included hardware was secured out of the surgical window to ensure no interference with imaging.

Neuromonitoring program

**Figure 4:**
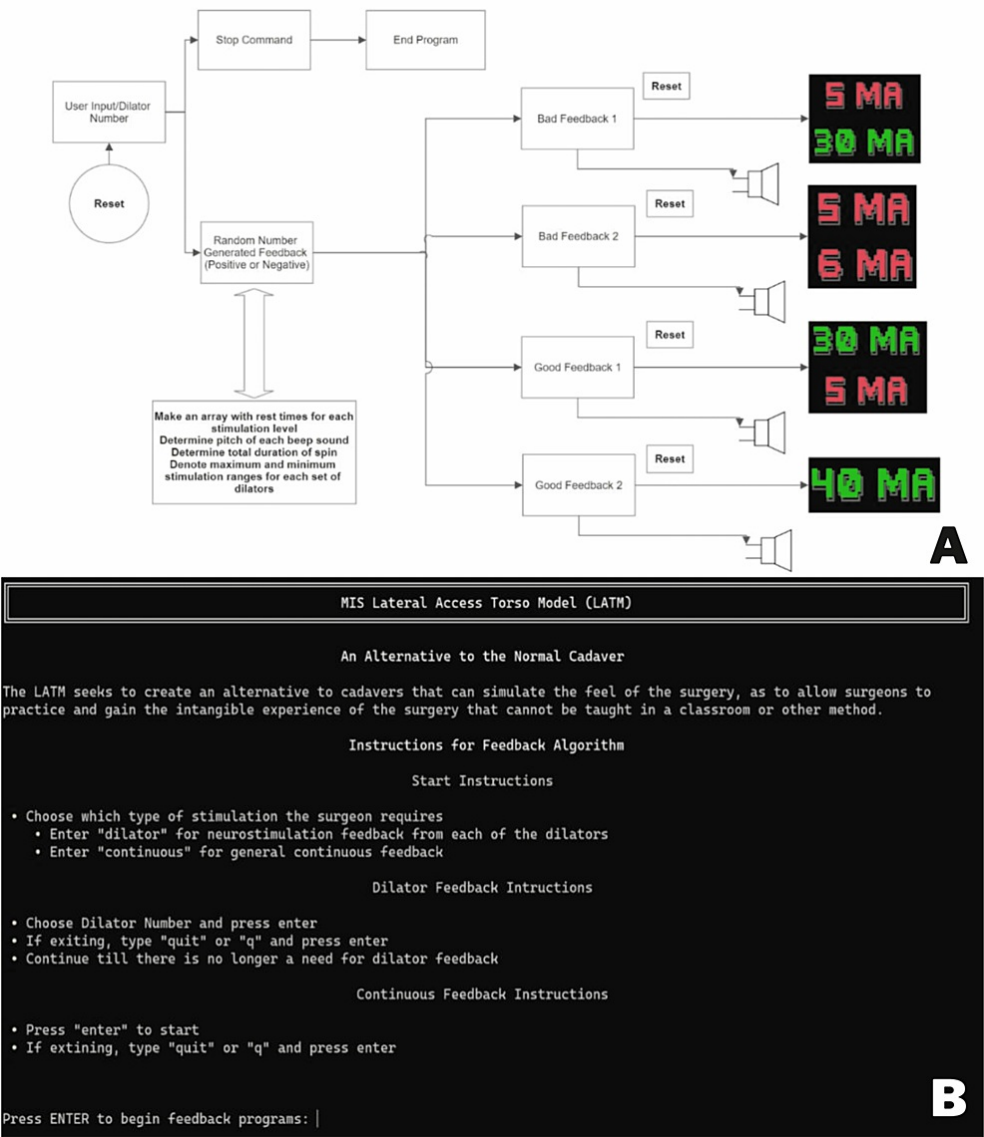
(A) Block diagram workflow of intraoperative neuromonitoring simulation algorithm. (B) Landing page of intraoperative neural monitoring training program complete with instructions.

**Figure 5:**
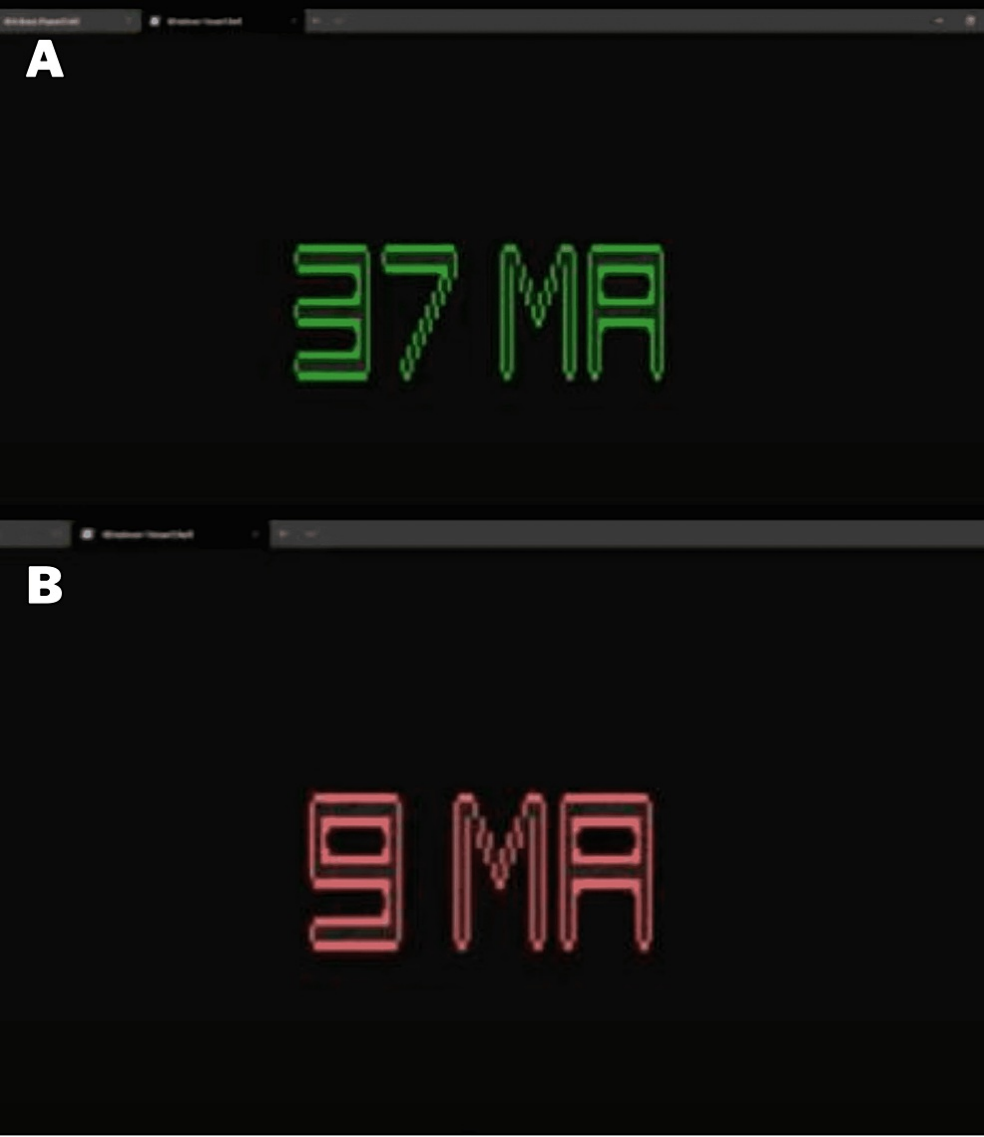
(A) Visual feedback from the intraoperative neural monitoring training programing indicating proper dilator position. (B) Visual feedback from the intraoperative neural monitoring training program indicating dangerous proximity to the lumbar plexus and prompting repositioning.

A core feature of the LATM is neuromonitoring feedback. A Python-based script was developed to provide random neuromonitoring data upon surgeon request (Figure 4A). The program can be accessed via command line/terminal and provides an overview on how to use the program (Figure 4B). The only software required is Python 3.7+, which is a free-to-download open-source coding program. As illustrated in the figure, various feedback is provided to the surgeon in a repeatable and convenient manner. This auditory and visual feedback is identical to actual neuromonitoring devices (Figures 5A, 5B).

Physical model

This simulator is designed to replicate a lateral retroperitoneal approach to the spine, that includes a minimally invasive approach directed to the lumbar vertebrae, dilator introduction, endoscopic or direct surgical views, psoas muscle manipulation with simulated preservation of the lumbar plexus, discectomies, and interbody implant placement.

Validation of model translation

A descriptive slide deck was sent to 30 surgeons (15 U.S. and 15 international surgeons) at both academic and private institutions who regularly perform LLIF. Overall, 30 (100%) of 30 responded that they felt this simulator has value for the field, 29 (96.67%) of 30 responded that they would have used this simulator if they had access during their training, and 30 (100%) of 30 responded they would encourage residents and medical students to train on such a simulator. Suggestions for future iterations included the addition of vasculature, integration of electronic components into the psoas, and inclusion of spinal deformities. Radiographic testing of the LATM was performed at T3 Labs (Atlanta, GA, USA), and the Mayo Clinic Simulation Center (Jacksonville, FL, USA). Accuracy and authenticity of the images were confirmed by consulting physicians.

## Discussion

LLIF is a relatively recent development in spine surgery, providing minimally invasive access for indirect nerve decompression, interbody fusion, and correction of lumbar degenerative deformity [14-16]. This technique represents a safe alternative to posterior spinal approaches and has gained acceptance among surgeons over the last decade due to favorable patient outcomes, as evidenced by radiographic parameters and clinical improvement [15,16]. Gaining surgical proficiency using this approach is challenging, and appropriate resident exposure to these cases is highly variable between programs and countries. Available alternatives to emulate the live experience in the operating room, such as cadaveric tissue, may constitute economic, religious, or regulatory conflicts, exemplifying the need for advanced versions of the proof-of-concept LATM.

The potential market for an LLIF surgery simulator is sizable and growing. Within the United States, 915 potential spine surgeons graduated in 2018 (186 neurosurgical residents, 729 orthopedic residents) compared to 782 (147 neurosurgery residents, 635 orthopedic residents) in 2009 [17]. Paralleling the growth in surgeons has been an increase in elective lumbar fusion procedures, from 122,679 in 2004 to 199,140 in 2015 (62.3% growth) [18]. Interestingly, the vast majority of additional cases were indicated for the treatment of spondylolisthesis, which may suggest a permanent increase due to the aging population in the US [18]. Furthermore, significant advancements in technology and surgical skills have influenced trends in spine surgery, driving surgeons and patients to favor minimally invasive interventions [4,5,16,19-21]. With clear growth in the popularity of minimally invasive spine surgery, it is a reasonable assumption that the need for training has increased as well. Prior investigations have shown the national average cost for cadavers to be $2,000, though more recent articles have shown the expense to be upward of $5,000 to $10,000 [22]. Given this, it is apparent that low-cost alternatives such as the LATM need to be further developed and put into practice.

A thorough prior art search found minimal commercial and academic attempts to create training tools for lateral access spinal procedures. A patent search led to the identification of eight surgical trainers of interest [23-30]. While all these products boast a level of realism, they differ between the specific procedures and conditions they are designed to teach. None of the identified simulators was inexpensive or explicitly designed to train minimally invasive lateral procedures.

Through research and interviews, it was determined that many areas of the world do not have access to cadavers for financial, religious, or social reasons. Moreover, synthetic models on the market are often priced at thousands of dollars, severely limiting global accessibility of training. The LATM needed to be constructed at low cost and require little to no maintenance. This model only incorporated components that could be stored at room temperature for extended periods of time. While this increased the difficulty of construction, it was done to ensure feasibility of storage and transport in an educational setting. The total cost of LATM was approximately $500. Most of the costs were a direct result of third-party manufactured components such as the outer torso shell and synthetic tissues. When manufactured at a large scale, it is estimated that this model will have a total raw material cost of under $100.

To determine the best materials to use in the spine module, multiple sets of lumbar vertebrae were printed in varying polymeric materials (ABS, PVC, PLA, etc.) and subsequently imaged. Physician feedback was given based on radiographic findings. Fluoroscopic imaging showed that ABS had the most accurate radiopacity when compared to organic bone. Tactility and haptic properties of the vertebrae were evaluated in a similar manner of physician confirmation. Previous work at the Mayo Clinic on analogous bone-like polymers further validated the team’s material selection.

The random neuromonitoring feedback program was pursued for two reasons. First, integration of electronic components into a nonconductive material such as silicone is not feasible. Therefore, creative solutions need to be developed that can work around this, and our laboratory is currently looking for a feasible answer. Second, this preliminary model is intended to teach the basics to junior residents, such as awareness of neurological complications and dilator repositioning. The LATM is not expected to have practical use for attending physicians in its current form, but future iterations may.

Study limitations

One of the objectives of this investigation was to put a spotlight on the lack of viable LLIF training alternatives. The created model is not intended or presented as a complete training device but rather a proof-of-concept; we acknowledge that the LATM lacks some key characteristics. Relevant intraoperative features such as the presence of crucial vascular elements, nerve roots and plexus, retroperitoneal fat, and ligaments represent an opportunity to improve the realism and the educational value of this simulator. We note that virtual evaluation of such a model is less than ideal, though the primary objective of this study was to propose an alternative method to train the basics of a complex procedure and not as a high-fidelity analog. Restrictions put in place due to the COVID-19 pandemic limited our ability to test in person.

## Conclusions

The LATM is a novel proof-of-concept design. The possible utility of this product is shown in the rising demand for physician training in lateral access procedures and the shortage of training assets. This was further enforced by the unanimous positive feedback we received from experts in the field looking for better ways to develop surgical skills in trainees. Future iterations of this model can impact individualized medicine, especially in financially limited countries, by providing inexpensive, realistic, and customizable components. This highly specific practice can lead to increased patient safety and improved outcomes. It also presents the opportunity to develop a recurrent revenue stream using the model to provide custom patient anatomy and replacement components. Next-generation versions of this model will build on the principles explored in this investigation.
